# Accessory Soleus Muscle: Two Case Reports with a Completely Different Presentation Caused by the Same Entity

**DOI:** 10.1155/2020/8851920

**Published:** 2020-09-14

**Authors:** Mihovil Plečko, Igor Knežević, Damjan Dimnjaković, Mario Josipović, Ivan Bojanić

**Affiliations:** ^1^Department of Orthopaedic Surgery, University Hospital Centre Zagreb, Šalata 6-7, 10 000 Zagreb, Croatia; ^2^School of Medicine, University of Zagreb, Šalata 2, 10 000 Zagreb, Croatia

## Abstract

Accessory soleus muscle (ASM) is a rare supernumerary anatomical variant that commonly presents as a posteromedial ankle swelling, which may become painful during physical activity. As it may mimic a soft tissue tumor, it is essential to differentiate this condition from ganglion, lipoma, hemangioma, synovioma, and sarcoma. However, ASM may also present with a painful syndrome, characterized by pain and paresthesia of the ankle and foot, mimicking the tarsal tunnel syndrome (TTS). Two cases of ASM are presented in this article. The first case had a typical presentation with painful posteromedial ankle swelling. After the initial assessment, the diagnosis was confirmed by magnetic resonance imaging (MRI), and ASM was treated by complete resection. The second case presented with pain and paresthesia in the right ankle and foot, but no swelling was noticeable. It was initially misdiagnosed by a rheumatologist and afterward overlooked on an MRI by a musculoskeletal radiology specialist and therefore mistreated by numerous physicians before being referred to our outpatient clinic. After further assessment, the diagnosis has been confirmed, and ASM was treated by complete resection combined with tarsal tunnel decompression. To the best of our knowledge, this is the first case reported in which ASM caused symptoms but presented without posteromedial swelling. This might be due to a proximally positioned belly of the ASM, followed by a tendinous insertion on the medial side of the calcaneus.

## 1. Introduction

Accessory soleus muscle (ASM) is a rare supernumerary anatomical variant that was first described by Cruveilhier [1] in 1843. As most of the accessory muscles, they are usually asymptomatic and incidentally discovered during radiographic imaging studies [2]. The incidence of ASM in the population is ranging from 0.7% to 5.5%. It occurs bilaterally in 15% of cases and is almost twice as common in males [3]. ASM most commonly presents as a posteromedial ankle swelling, which may become painful during physical activity [4]. In other cases, it may present as painless swelling and rarely associated with clubfoot or equinus deformity [3]. As it may mimic a soft tissue tumor, it is essential to differentiate this condition from ganglion, lipoma, hemangioma, synovioma, and sarcoma [5]. ASM may also present with a painful syndrome, characterized by pain and paresthesia of the ankle and foot, mimicking the tarsal tunnel syndrome (TTS) [6]. Here, we report on two symptomatic cases of ASM, one with a typical presentation and treatment protocol and one with an atypical presentation, which led to delayed diagnosis and treatment. The patients agreed to the publication of the data and accompanying images concerning these cases.

## 2. Case Report One

**Figure 1:**
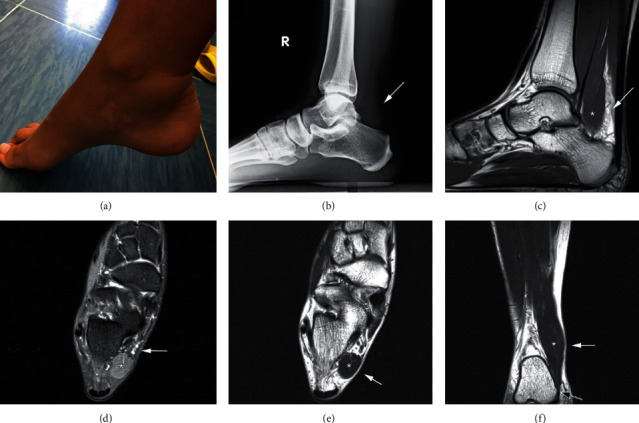
Preoperative images of the patient's right ankle presented in the first case. (a) A swelling is visible in the posteromedial part of the right ankle, which is more prominent in plantar flexion. (b) Obliteration of Kager's fat pad visible on the plain lateral radiograph of the right ankle is pointed by a white arrow. (c) T1-weighted sagittal MR image of the right ankle. Accessory soleus muscle (ASM) is marked with an asterisk (^∗^), and obliteration of the Kager's fat pad is pointed by a white arrow. (d) T2-weighted axial MR image of the right ankle. ASM is marked with an asterisk (^∗^), and neurovascular bundle in the posteromedial part of the ankle is pointed by a white arrow. (e) T1-weighted axial MR image of the right ankle. ASM is marked with an asterisk (^∗^), and elevation of the skin and subcutaneous tissue is pointed by a white arrow. (f) T1-weighted coronal MR image of the right ankle. ASM is marked with an asterisk (^∗^), the elevation of the skin and subcutaneous tissue is pointed by a white arrow, and muscular insertion of the ASM on the medial side of the calcaneus is pointed by a black arrow.

**Figure 2:**
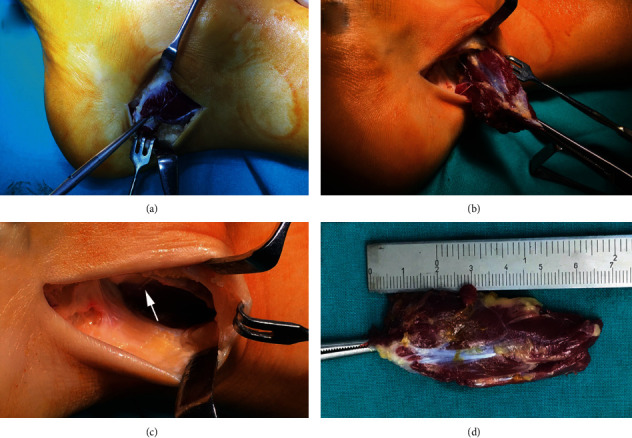
Intraoperative images of the patient presented in the first case. (a) Incision was made just medial to the Achilles tendon proximally and extended down to the proximal border of the tarsal tunnel. Accessory soleus muscle (ASM) is visible right below the skin and subcutaneous tissue. (b) ASM was bluntly dissected and separated from the surrounding structures. (c) Neurovascular bundle of the posteromedial part of the ankle is visualized. Neurovascular bundle is pointed by a white arrow. (d) The completely resected muscle that is approximately 7 cm long. A muscular distal portion is marked by a Kocher's forceps.

A 25-year-old female with pain and swelling of the right ankle and no history of trauma reported to our outpatient clinic. Past medical history was unremarkable. Eight years ago, she noticed a painless posteromedial ankle swelling. Four years ago, she noticed occasional pain and increased swelling of the ankle during and after running, which resolved with rest. Over time, the pain started occurring while walking and occasionally during the night, which would wake her up from sleep. The American Orthopaedic Foot & Ankle Society (AOFAS) Ankle-Hindfoot score was 48, while visual analogue scale (VAS) for pain was 3 while resting and 8 in movement. Physical examination revealed painful posteromedial ankle swelling (Figure 1). Standing up on toes provoked pain in the right ankle. There were no noticeable skin changes. The normal range of motion in the ankle was observed, with no signs of ankle instability or impingement. There were no signs of neurovascular impairment, with negative Tinel's sign. On plain lateral radiographs, the obliteration of the Kager's fat pad was observed, with no bony deformities or malalignment. On MRI, the presence of ASM was revealed, which had a muscular insertion on the medial side of the calcaneus. As the symptoms progressed, surgery was scheduled. The procedure was performed under spinal anesthesia in a supine position with the use of a tourniquet. The incision was made just medial to the Achilles tendon proximally and extended down to the proximal border of the tarsal tunnel (Figure 2). ASM was identified, bluntly dissected, and completely resected. Histopathologic analysis confirmed that the resected specimen was a skeletal muscle. Non-weight-bearing walk with two crutches was recommended for the first two weeks. After that, weight-bearing, as tolerated, was allowed in conjunction with recommended range-of-motion exercise of the ankle. Gradual return to daily activities was permitted ten weeks after the surgery. At the final follow-up six months after the surgery, the patient showed significant improvement with no complications or recurrence of symptoms and has returned to her previous level of activity. The AOFAS Ankle-Hindfoot score was 90, while VAS for pain was 0 while resting and 2 in movement.

## 3. Case Report Two

**Figure 3:**
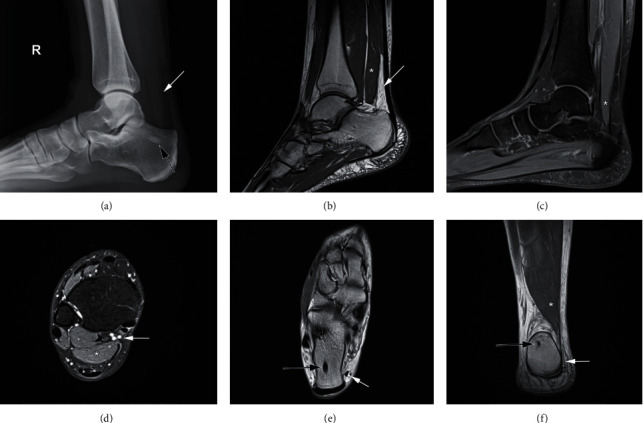
Preoperative images of the patient's right ankle presented in the second case. (a) Obliteration of Kager's fat pad visible on the plain lateral radiograph of the right ankle is pointed by a white arrow, and insula compacta is pointed by a black arrow. (b) T1-weighted sagittal MR image of the right ankle. Accessory soleus muscle (ASM) is marked with an asterisk (^∗^), and obliteration of the Kager's fat pad is pointed by a white arrow. (c) T2-weighted sagittal MR image of the right ankle. ASM is marked with an asterisk (^∗^), where shrinkage of the muscle can be visualized towards its insertion. (d) T2-weighted axial MR image superior to the right ankle. ASM is marked with an asterisk (^∗^). In this plane, the muscle is positioned closest to the neurovascular bundle that is pointed by a white arrow. (e) T1-weighted axial MR image of the right ankle. Tendinous insertion of the ASM on the medial side of the calcaneus is pointed by a white arrow, and insula compacta in the calcaneus is pointed by a black arrow. (f) T1-weighted axial MR image of the right ankle. ASM is marked with an asterisk (^∗^), tendinous insertion of the ASM on the medial side of the calcaneus is pointed by the white arrow, and insula compacta in the calcaneus is pointed by a black arrow. No elevation of the skin and subcutaneous tissue is visible.

**Figure 4:**
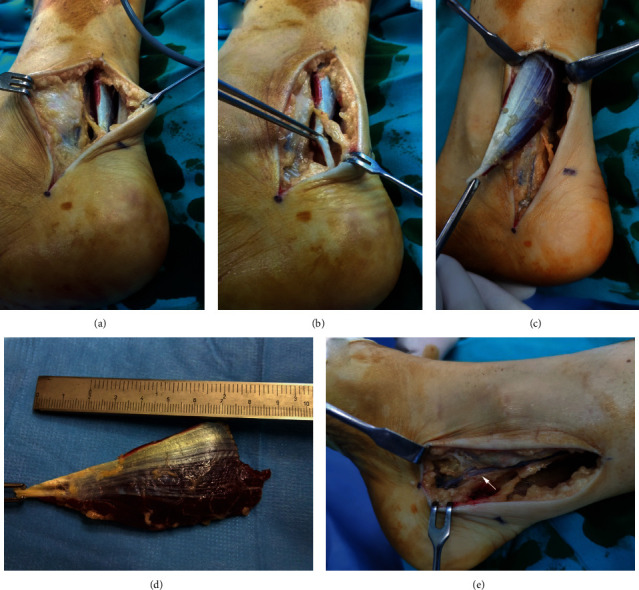
Intraoperative images of the patient presented in the second case. (a) Incision was made just medial to the Achilles tendon proximally and extended down to the proximal border of the tarsal tunnel. Accessory soleus muscle (ASM) is visible right below the skin and subcutaneous tissue. (b) ASM is elevated by a surgical pincer. Shrinkage of the muscle can be visualized towards its insertion. (c) ASM was bluntly dissected and separated from the surrounding structures. (d) The completely resected muscle that is approximately 8.5 cm long. A tendinous distal part is marked by a surgical pincer. (e) Neurovascular bundle of the posteromedial part of the ankle is visualized after resection of ASM and the flexor retinaculum. Neurovascular bundle is pointed by a white arrow.

A 31-year-old female with pain in her right ankle and paresthesia along the medial plantar aspect of the foot reported to our outpatient clinic. No history of trauma was reported. Last 8 years, she was regularly monitored by a rheumatologist due to Raynaud's syndrome. Since the ankle symptoms started about a year ago, she significantly gained weight due to medications and inactivity. She was initially examined in an emergency department regarding her ankle pain. No definitive diagnosis was set at the time, and she was referred to a rheumatologist for further assessment. Her symptoms persisted after the initial conservative therapy with NSAIDs and rest. She was further treated with high doses of intravenous corticosteroids despite unremarkable capillaroscopy and regular laboratory work-up for immune diseases. As the pain persisted, sulfasalazine was added to therapy with no effect on symptoms. She was further referred for a three-phase bone scan and ultrasound (US) of the ankle, which were also unremarkable. At that moment, she was diagnosed with complex regional pain syndrome, and lidocaine patches were added into therapy. In the meanwhile, she was referred to a physical therapy specialist, and physical therapy for alleviation of pain was scheduled. Rheumatologist referred her for an MRI of the right ankle, which was reported as unremarkable by a musculoskeletal radiology specialist. As symptoms persisted, she was further referred to a pain management clinic and for an orthopedic surgeon's examination. Opioids and pregabalin were introduced into therapy, and the patient was referred for hyperbaric chamber therapy. Following hyperbaric chamber therapy, she noticed an improvement in sensation in her foot while ankle pain persisted. In the meantime, she presented to our outpatient clinic, where an orthopedic surgeon issued a plain radiograph of the right ankle, and a dense area was noted in the right calcaneus (Figure 3). Requested computerized tomography (CT) scan of the right ankle showed insula compacta (10 × 4 mm) in the calcaneus, with no other remarkable findings. At that point, an experienced foot and ankle specialist reviewed the case. The AOFAS Ankle-Hindfoot score was 18, while VAS for pain was 10 while resting and 10 in movement. Physical examination revealed a limited range of motion in the ankle due to pain but without signs of ankle instability or impingement. Standing up on toes was not possible due to pain. The posteromedial part of the ankle was painful on palpation. A positive Tinel's sign was elicited on percussion of the tibial nerve proximal to the tarsal tunnel. This recreated her posteromedial ankle pain and also elicited numbness and tingling along the medial plantar aspect of the foot. On a plain radiograph, the obliteration of the Kager's fat pad was observed. Orthopedic surgeon analyzed the earlier mentioned MRI and made a provisional diagnosis of ASM, which was later confirmed by a more experienced musculoskeletal radiology specialist. ASM had a long tendinous insertion on the medial side of the calcaneus. As there was high suspicion for concomitant TTS, the patient was referred for electromyoneurography (EMNG), which confirmed the diagnosis, and surgery was scheduled. The procedure was performed under spinal anesthesia in a supine position with the use of a tourniquet. The incision was made just medial to the Achilles tendon proximally and extended down to the proximal border of the tarsal tunnel (Figure 4). ASM was identified, bluntly dissected, and separated and resected completely. The flexor retinaculum was released in its entire course, and the tibial nerve was mobilized with resection of fibrous tissue. Further, the superficial fascia on the abductor hallucis was released, the muscle was retracted, and a complete release of tunnels for medial and lateral branches of the nerve was made with the removal of the central fibrous septum. Histopathologic analysis confirmed that the resected specimen was a skeletal muscle. Non-weight-bearing walk with two crutches was recommended for the first two weeks. After that, weight-bearing, as tolerated, was allowed in conjunction with recommended range-of-motion exercise of the ankle. Gradual return to daily activities was permitted ten weeks after the surgery. At the final follow-up six months after the surgery, the patient showed significant improvement with no complications or recurrence of symptoms and has returned to her previous level of activity. The AOFAS Ankle-Hindfoot score was 100, while VAS for pain was 0 while resting and 0 in movement.

## 4. Discussion

Accessory muscles of the ankle should be included in the differential diagnosis of chronic ankle pain. Although congenital, ASM is usually diagnosed in the second or third decade of life when symptoms occur. During this period, muscle mass and physical activity increase, more prominently seen in male patients [7, 8].

There are several theories to what causes pain in patients with ASM. According to some, enlargement of ASM during exercise is the cause of a localized compartment syndrome which is relieved with rest. Others point to the theory that during exercise, ASM might be insufficiently supplied by blood from the posterior tibial artery, thus producing true claudication which is relieved with rest. Moreover, symptoms might be a result of compression neuropathy due to the proximity of ASM to the tibial nerve [9]. While the cause of symptoms in our first case might be found in the former two theories, in our second case, symptoms could also be explained by the third theory.

ASM is usually covered by its fascia and gets neurovascular supply from the tibial nerve and the posterior tibial artery [9]. It may originate from the fibula, soleal line of the tibia, or the anterior surface of the soleus muscle. Its insertion can be either muscular or tendinous [2]. Lorentzon and Wirell [10] initially described four insertions: on the distal part of the Achilles tendon, muscular or tendinous insertion on the superior side of the calcaneus, and muscular insertion on the medial side of the calcaneus. Yu and Resnick [11] later described a tendinous insertion on the medial side of the calcaneus. In the first case, a muscular insertion on the medial side of the calcaneus was present, while in the second case, a tendinous insertion on the same area was seen. The second patient did not present with a posteromedial ankle swelling, which might be due to a more proximally positioned belly of the ASM. To the best of our knowledge, this is the first case reported in which ASM caused symptoms but presented without posteromedial swelling.

Kinoshita et al. [12] observed that TTS triggered by compression of ASM, although considered unusual, was present in 4.1% of cases in their study. However, a growing number of recent case reports implicating accessory anatomy as the etiology for compression at the tarsal tunnel suggest this may not be as rare as it was thought. Neary et al. [13] consider this is due to increased implementation of an ankle MRI in routine diagnosis and increased surveillance for accessory muscles in that region. Doda et al. [7] state that the key to diagnosing ASM is registering an MRI signal that is typical for a well-encapsulated skeletal muscle situated in an atypical anatomic location.

Diagnostic imaging usually starts with plain radiographs, where obliteration of Kager's fat pad might be observed. As Kendi et al. [4] report, this is not pathognomonic, but can be highly suggestive for the diagnosis of ASM. Furthermore, US and CT might be useful in the assessment, but definitive diagnosis may be set only by an MRI. Our second case makes clear the importance of having a high degree of suspicion for the presence of accessory muscle when interpreting MRI results as part of the diagnostic work-up.

Severity of symptoms determines the method of treatment. If the patient is asymptomatic, no further therapy is needed. However, if the patient complains of pain, conservative management such as activity modification, physical therapy, and use of NSAIDs is an option [7]. Furthermore, the application of botulinum toxin type A into ASM has been described as an effective treatment option with the goal to reduce muscle mass and tone [14]. However, in some cases, this therapy proved to relieve symptoms for a shorter period, possibly being insufficient in long-term management [14]. Therefore, surgery should be considered. Ligation of the irrigating artery, tendon release, fasciotomy, and partial and complete resection were described [15–17]. So far, only one case of ligation of the irrigating artery for ASM was reported, resulting in atrophy of the muscle [17]. Also, one case of a minimally invasive surgical resection of ASM tendon was performed in an athlete, where a rapid return to activity was desired [18]. However, this technique might lead to the recurrence of symptoms. Reddy and McCollum [9] state that studies showed fasciotomy and resection to be equally effective. Furthermore, they propose fasciotomy for patients with a small ASM and with a low level of activity, while for those with a large ASM and a high level of activity, they propose resection [9]. On the other hand, Kouvalchouk et al. [19] studied 21 cases of ASM, and suggest that if surgical treatment is needed, complete resection is preferred over fasciotomy. Similarly, Rossi et al. [20] prefer resection over fasciotomy because even after fasciotomy, a voluminous muscle might put compression on neurovascular structures. Therefore, we decided to perform a complete resection of ASM in both of our cases. As our second case also presented with TTS, appropriate management was needed. Kinoshita et al. [12] reported on a case with TTS caused by ASM, in which they did a complete decompression of the tibial nerve combined with resection of ASM. Neary et al. [13] presented a case in which the patient had refractory symptoms caused by tibial nerve compression. In that patient, both flexor digitorum accessories longus and ASM were identified and completely resected, followed by a tibial nerve decompression [13]. Because of severe symptoms caused by TTS, we decided to include the usual method we use for tarsal tunnel decompression for the second patient.

In conclusion, ASM should be included in the differential diagnosis when a patient presents with posteromedial ankle pain, even if no swelling is initially observed. It is vital to recognize ASM on an MRI, which may prevent the development of chronic pain syndrome. Also, we recommend complete resection of ASM in all symptomatic patients, combined with tarsal tunnel decompression in patients that show concomitant symptomatology typical for TTS.
